# Prioritising physical and psychological symptoms: what are the barriers and facilitators to the discussion of anxiety in the primary care consultation?

**DOI:** 10.1186/s12875-019-0996-6

**Published:** 2019-07-27

**Authors:** M. C. Barnes, D. Kessler, C. Archer, N. Wiles

**Affiliations:** Centre for Academic Mental Health, Population Health Sciences, Bristol Medical School, University of Bristol, Bristol, UK

**Keywords:** Anxiety, Primary care, Qualitative

## Abstract

**Background:**

Anxiety is under-recorded and under-treated in the UK and is under-represented in research compared with depression. Detecting anxiety can be difficult because of co-existing conditions. GPs can be reluctant to medicalise anxiety symptoms and patients can be reluctant to disclose them, for a variety of reasons. This research addresses the gap in evidence of real-life consultations of patients with anxiety and explores how physical and psychological symptoms are discussed and prioritised by patients and GPs in primary care consultations.

**Methods:**

A mixed methods study using a baseline questionnaire, video-recorded primary care consultations and interview data with patients and GPs.

**Results:**

Seventeen patients with anxiety symptoms (GAD-7 score ≥ 10) completed a questionnaire, had their consultation video-recorded and took part in a semi-structured interview. Four GPs were interviewed. The main themes that emerged from GP and patients accounts as barriers and facilitators to discussing anxiety mostly mirrored each other. The GP/patient relationship and continuity of care was the main facilitator for the discussion of anxiety in the consultation. The main barriers were: attribution of or unacknowledged symptoms; co-morbidities; and time constraints. GPs overcame these barriers by making repeat appointments and employing prioritising techniques; patients by choosing an empathetic GP.

**Conclusions:**

The findings add to the evidence base concerning the management of anxiety in primary care. The findings suggest that the discussion around anxiety is a process negotiated between the patient and the GP influenced by a range of barriers and facilitators. Co-existing depression and health anxieties can mask anxiety symptoms in patients. Good practice techniques such as bringing back patients for appointments to foster continuity of care and understanding can help disclosure and detection of anxiety symptoms. Future research could investigate this longitudinally and should include a wider range of GPs practices and GPs.

**Electronic supplementary material:**

The online version of this article (10.1186/s12875-019-0996-6) contains supplementary material, which is available to authorized users.

## Background

Mental health problems represent a large proportion of the disease burden in the UK, with anxiety disorders accounting for 14.6% of burden in terms of disability-adjusted life years [1]. Most common mental disorders, including anxiety, are treated in primary care in the UK. The introduction of the Improving Access to Psychological Therapies (IAPT) service has meant that many more patients are offered psychological interventions. However, GPs retain overall responsibility for care and IAPT services do not prescribe medication.

Anxiety disorders are more chronic than other common mental health disorders [2] but rates of diagnosis and treatment are lower than expected [3]. The most recent Psychiatric Morbidity Survey reported that only 37% of people with anxiety and depression are accessing treatment [4] and the under-recording of anxiety disorders in clinical care is widely acknowledged [5]. There are a number of possible reasons for this. Anxiety disorders often co-exist with depressive disorders and long-term physical health conditions, making detection more difficult [6]. These co-morbid conditions have a worse prognosis than either anxiety or depression alone, with greater disability and high health and social costs [7–9]. Patients with anxiety and depression often present somatic symptoms to their GP and diagnosis of an underlying psychological disorder can be a process of excluding somatic illness and working with patients towards a reattribution of symptoms.

Although they may feel confident in recognising anxiety symptoms, GPs tend to prefer symptom codes to diagnostic anxiety codes as they often feel symptoms can be transient, or are concerned about medicalising life events [6, 10, 11] Patients are often reluctant to disclose mental health problems, including anxiety, because of concerns about stigmatisation, and a view that clinicians prioritise physical over mental health [12, 13].

Compared with depression, anxiety disorders are under-represented in UK primary care research. It is likely that this is due to both patient and practitioner factors [11]. Missing from the evidence is any examination of ‘real-life’ consultations involving patients with anxiety and how the experience and expectations differ between patients and practitioners. Therefore, the aim of this study was to examine how physical and psychological symptoms – in particular anxiety – are discussed and prioritised by patients and GP in consultations in order to better understand the barriers and facilitators involved in the primary care consultation.

## Methods

### Design

This mixed methods study collected data from several sources: a baseline questionnaire to identify patients of interest prior to their consultation in primary care; video recordings of the consultations; and semi-structured interviews with patients and GPs.

### Study population

Eligible participants were patients aged 18 years or over attending a GP appointment on specified recruitment days. GPs had a list of the exclusion criteria to use before invitation packs were sent out from the practice: they could not give informed consent, had a substance abuse issue as their main problem, had psychosis or were under 18. The researchers did not have access to this information as it was prior to participant’s giving consent.

We were not able to include patients who were not fluent in English as funding did not allow for translation services.

### Recruitment

GP Practices in Bristol were sent a summary of the research proposal and responded with expressions of interest. The first practices representing a higher and lower socioeconomic demographic were approached and all GPs within the two practices were invited to take part. No authors were involved in any of the patient care.

Study information and invitation letters were sent to patients up to 3 weeks prior to their consultation, following review of eligibility by the appropriate GP.

On recruitment days written consent for recording and possible follow-up interview was taken by the main researcher (MB) or member of the Clinical Research Network (CRN). The patient completed a baseline questionnaire prior to their appointment. This included socio-demographic details, reason for their visit together with measures of depression (PHQ-9), health anxiety (HAI), physical co-morbidity, general health, and measure of anxiety (GAD-7). The GAD-7 is a 7-item scale for evaluating the presence and severity of anxiety. A score of ≥10 indicates moderate to severe levels of anxiety [14].

For the purposes of this project, anxiety was defined as self-reported severity of symptoms in the GAD questionnaire across 7 domains: nervousness, control of worrying, worrying about different things, trouble relaxing, restlessness, becoming easily annoyed and feeling afraid as if something awful might happen.

Following completion of the questionnaire, the GP appointment was carried out as usual. With consent, consultations were video-recorded.

Patients who scored 10 or more on the GAD-7 were contacted by the researcher to arrange a convenient time for interview. All video-recordings with patients with GAD-7 scores< 10 were deleted.

### Interviews

Participants were interviewed face-to-face by MB in the patient’s home or a room in the GP practice. Interviews were audio recorded and a topic guide was developed from previous literature [10, 15, 16, 18] and discussions with service user research advisors. Participants were asked questions about their recent discussion with their GP and history of anxiety.

GPs were interviewed at the end of the recruitment period in their practice by MB and were asked questions about their diagnostic process. The topic guide was developed with reference to the previous literature, and discussion with the study team that included a GP (Additional file 1). Topic areas included: techniques used to identify and prioritise physical or psychological symptoms; and how symptoms are managed. GPs were also shown a video clip of one of their consultations with a patient with anxiety symptoms (GAD-7 score ≥ 10) and asked to talk about the consultation and any barriers or facilitators to discussing anxiety.

The interviews were transcribed verbatim by a member of University staff and data was organised using NViVO 10 qualitative software.

### Analysis

#### Interview data

Data collection and analysis occurred concurrently and iteratively, cross-sectionally and in case-studies, according to the constant comparison methods of grounded theory [15]. Data relating to the first four interviews were analysed by detailed scrutiny of the transcripts to identify common themes which were then coded (MB). A coding comparison exercise then took place with other members of the research team (NW/DK). Codes were refined and the framework used to code the rest of the transcripts. GP data were analysed similarly. Data were examined for similarities and differences within themes and across sets i.e. consultations where anxiety symptoms were discussed or not. Analysis of all data was pursued to saturation.

#### Video data

For the purpose of this analysis, video recordings were used to categorise patient consultations into those where anxiety was and was not explicitly discussed. Further analysis relating to the actual content of consultation will be reported separately.

## Results

In total, 160 participants (82 women, 52%; mean age 53.4 years (SD 19.6) (Table 1) consented to having their consultation recorded and completed the questionnaire. Twenty-one participants had a GAD-7 score of 10 or more, of whom 17 were interviewed (two participants were lost to follow up and two participants had a consultation recording error). Of the 17 participants, one was a person of colour and 3 spoke English as a second language. The research team approached all eligible patients booked to see the GP on the recruitment day. Of those approached, 59 (27%) actively declined to take part in the study and 160 (73%) agreed to participate.

**Table 1:** Sociodemographic characteristics and consultation data of study participants

	All patients	Those interviewed
	(n = 160)	(n = 17)
Age in years: mean (SD)^a^	53.4 (19.6)	47.4 (16.8)
Female: n (%)^b^	82 (52.2)	10 (58.8)
Reasons for consultation: n (%)^d^		
To find out what is wrong/get diagnosis	80 (50)	7 (41.2)
For reassurance	27 (16.9)	5 (29.4)
To get the results of test/investigations	28 (17.5)	6 (35.3)
For treatment (prescriptions/procedures)	50 (31.3)	10 (58.8)
For a routine check	15 (9.4)	2 (11.8)
For review	29 (18.1)	2 (11.8)
To ask for a referral	17 (10.6)	0 (0)
Other	12 (7.5)	0 (0)
Consulting for more than one reason: n (%)	66 (41.3)	8 (47.1)
First time consulted doctor for this problem: n (%)	61 (38.1)	3 (17.7)
Previously diagnosed anxiety disorder (incl. Anxiety/depression)^c^	Not available	7 (41.2)
How long had problem consulting about: n (%)		
1 week or less/More than 1 week but less than 1 month	22 (13.8)	0 (0)
1 month or more, but less than 6 months	36 (22.5)	2 (11.8)
6 months or more	88 (55)	14 (82.4)
Not applicable	14 (8.6)	1 (5.9)
PHQ-9 score: median (IQR)^e^	3 (1, 6)	12 (10, 16)
GAD-7 score: median (IQR) ^f^	2 (0, 5)	14 (11, 16.5)
GAD-7 score ≥ 10: n (%)^f^	20 (13)	17^g^ (100)
EQ-5D utility score: mean (SD)^f^	0.74 (0.22)	0.6 (0.22)
Health Anxiety score: mean (SD)^h^	2.6 (2.1)	4.3 (2.7)
Any long-standing health condition: n (%)	92 (58.2)	12 (70.6)

Not all participants completed all items on the questionnaire, number with data where incomplete given below:

^a^ n = 155

^b^ n = 157

^c^ = from previous 12 months GP notes

^d^ Participants could choose multiple answers for this question, hence numbers will not add up to expected total

^e^n = 144 (n = 14 for those interviewed)

^f^ n = 154 (n = 16 for those interviewed)

^g^One interviewee had a GAD-7 score > =10, but did not complete all items on the GAD-7

^h^ n = 152 (n = 15 for those interviewed)

Those interviewed were, on average, aged 47.4 years (SD 16.8) and the majority (n = 10; 59%) were female. The median GAD-7 and PHQ-9 scores were 14 and 12 respectively (Table 1). Many (n = 12, 70.6%) reported a long-standing health condition, frequently asthma, diabetes, high blood pressure, back problems or mental health problems (Table 1). The most common reasons for consulting their GP were: to find out what was wrong or to get a diagnosis (41%); to get the results of tests (35%); or to get treatment (59%).

Four GPs from the two participating surgeries were interviewed. One doctor was female, three were in their thirties and one was 50 years old. They had been consulting in general practice between 6 and 25 years.

Patients with anxiety were evenly distributed amongst the GPs. Patients from practice 1 were more likely to report a more ongoing relationship with their GP (Additional file 2).

Video findings showed that within consultations, discussion and prioritisation of anxiety between the GP and patient was co-constructed in a number of ways: open discussion by both parties [OD]; a more elliptical or implicit discussion where it may be mentioned by one party only or where only medication is discussed [ID]; or not discussed at all [ND].

The main themes and sub-themes that emerged from interview accounts as barriers or facilitators around prioritising and discussing anxiety within consultations are shown in Table 2 and, largely, mirrored each other. Mirrored themes are presented together in the text under patient/GP titles. Themes specific to the patient or GP findings are described separately at the end of each section. Relevant observation data from the video consultations will be reported alongside the themes. All names for evidential quotes have been changed to preserve anonymity.

**Table 2:** Barriers and facilitators to discussing anxiety

	Patient Theme	GP Theme
Facilitator	Relationship with GP	Continuity of Care
Barriers	Attribution of symptoms - Aetiology - Social determinants	Unacknowledged symptoms
	Co-morbidities	Co-morbidities
	Time constraints	Stigma
	Gender/age	Time constraints
Techniques to Overcome Barriers	Choosing a Sympathetic GP	Repeat appointments
		Prioritising techniques

### Facilitating discussion

#### Relationship with GP/continuity of care

Most patients, from all groups, had positive things to say about their GP to do with being heard, cared for and exploring options together, for example:

*Brilliant, very attentive, really listening* (Female 65–75 ID)

*Very understanding, nice bloke* (Male 65–75 ID)

*he was very good and he’s got a very good manner which leads me to believe… that he genuinely cares and he’s got consideration, thought about not just the immediacy, like raising the CBT, looking at other avenues and things, the next step, so whenever I can I try and get him, and the fact that he’s always busy…* (Male 35–50 OD)

Being able to discuss anxiety was not simply to do with whether or not the GP, and the doctor/patient relationship, is considered ‘good’; there are other influential factors involved.

For GPs an important facilitator towards identification, discussion and management of anxiety was the continuity of the patient relationship.

*I would say one of the benefits of good general practice is hopefully the continuity of care, you get to know your patients and you get to know what normal is for them, what are the issues in their life going on…gradually over time and many meetings you get to know all of that so they’re more familiar with you and able to come out and say exactly what they’re worried about and also you can pick up when something’s not right…prioritising continuity is really important.* (GP4)

Continuity was viewed as benefitting both sides of the relationship and aiding open discussion

*Maybe that’s not why they came in but I think if you don’t build up that relationship then that’s a real loss to both parties isn’t it? So I think its experience of getting to know people and figuring out where they’re coming from and sometimes it can be implicated* [in anxiety]. (GP2)

Conversely, any factors which threaten continuity can be a barrier, such as the requirements of the younger patients and structural factors:

*A lot of patients do see the value in continuity but increasingly - the younger population it’s more about the convenience of getting the appointment sooner and they don’t necessarily care about who that is… It’s something we’re really wary of having merged because although it gives you a greater range of GPs and appointments it’s also a greater threat to that continuity…so you can end up with patients being seen by lots of different people being potentially over-investigated and not getting to the nitty gritty of what’s going on in their life,* [what's] *making them overly anxious* (GP4)

### Barriers to discussion

#### Attribution/unacknowledged symptoms

Many physical symptoms may be attributed to anxiety by both doctor and patient (Additional file 3). Participants attributions influenced when and if they openly discussed anxiety with their GP. For some, the attribution was straightforward but for others, experiencing physical symptoms for a condition viewed as a mental health problem could cause uncertainty:

*A couple of years ago I never had anxiety at all and I actually struggled to understand how people got it because I thought it was mostly a mental thing but the past year I’ve had anxiety and it’s a lot worse than I thought it would be, it’s like physical as well* (Female 20–35 ND)

Participants’ lack of knowledge about anxiety – in comparison to their understanding of depression - could delay help-seeking, or symptoms are not recognized as anxiety:

*I didn’t even know what it was… I’d never really heard of hit. I knew what depression was very well* (Female 35–50 OD)

*I don’t get anxiety* (Female 35–50 ID)

How patients viewed the causes of their anxiety also affected the discussion. Normalisation was a common theme. Many participants who did not openly discuss their anxiety felt it was a part of their personality:

I: *When you saw the Dr the other day, you didn’t* [mention]*-*

P: *No ‘cos I just think it’s part of me I suppose. I don’t know if they could do anything about it. I’ve not really sat down and talked to a doctor about it* (Female 35–50 ND)

GPs described the difficulties in consultations with patients who do not acknowledge their symptoms as anxiety and can lead to repeat presentations:

*I think they’re probably the hardest ones aren’t they to pick up on because they don’t acknowledge it as a separate problem…and sometimes it’s just acknowledged by the doctor but the patient maybe doesn’t register that that’s why they keep presenting…* (GP2)

Timing is important: if anxiety is addressed directly by the GP and the patient is not ready to acknowledge it, they can be put off returning at all:

*I put it on the table and say ‘look anxiety is a problem too, I sense a lot of it here, would you be interested in coming back?’ So, I would almost insist that they think of it as a problem in its’ own right and if you want to come and talk to me about your anxiety, I’d really like to help and it’s amazing how few people come back…* (GP3)

It was recognised that lack of concordance between patient and GP interpretation of symptoms can be difficult for patients, but that it is part of the GP’s role to have a discussion with the patient about anxiety, if relevant:

*that’s frustrating for the patient if you have a GP who continually says ‘I think its anxiety’ so you don’t push it too hard but I think you need to… if you feel that that’s the main cause of the problem then you need to try and at least raise it as a possibility with the patient.* (GP4)

Structural factors also played a role in the aetiology of, and barrier to, discussions of anxiety. Anxiety often appeared to be a background noise in participants’ lives that would ebb and flow with circumstances. These circumstances could sometimes prove overwhelming and then the basic necessities of life were prioritised over addressing their symptoms:

*I you didn’t talk specifically to your doctor about anxiety or anything did you, on that consultation?*

*P Not on that one but I have before. Purely because I don’t know what’s happening, I don’t know what we’re going to do, it’s a case of if my wife loses her job we can’t even buy food let alone anything else ‘cos I’ve got no income whatsoever and we just live on her minimum wage but we’ve got nowhere to go in July,* (Male 50–65 ID)

The acknowledgment of the role of economic, political, structural and social factors as barriers were mirrored in GP accounts when describing how the context of patients’ lives could be an obstacle to managing symptoms:

*I think there’s a sort of resignation amongst GPs in every practice, particularly in (area) where there’s a lot of people who it doesn’t matter how hard you work with them, they’re buffeted on the wings of austerity, welfare support, lack of social care, a lack of hope there’s very little in the social network… in terms of supportive charities, government initiatives that will back anything you say up.* (GP3)

#### Co-morbidities

Most participants presented with another condition which could mask the anxiety (Additional file 4). Depression was the most common co-morbidity and most likely to be discussed with patients where anxiety was not talked about explicitly. Physical co-morbidity was most likely among those who did not discuss anxiety at all.

There was some overlap in accounts about anxiety and depression. Because management of depression and anxiety are similar, there was a further barrier to discussing anxiety symptoms as a different entity for patients and GPs:

*I do you see the anxiety, as separate from the depression or-?*

*P (long pause) I don’t know if it’s two different things or what. Maybe it’s because (pause) each person’s different. Maybe each person’s different.* (Female 50–65 ND)

*I mean maybe I don’t always distil out exactly the difference between anxiety and depression.* (GP1)

Health anxiety – the preoccupation with having a serious illness despite medical reassurance – could also mask generalised anxiety symptoms. Participants who also self-reported high anxiety scores could return many times to the practice if their expectations of positive test results were not met or they did not feel reassured by the GP:

* I mean I still get paranoid sometimes because…Especially when you’re reading on the internet all the symptoms about chronic pain and cough and stuff and what’s getting me most is they do tests and all tests come* [negative] (Male 20–35 ID)

Whilst health anxiety can mask more generalized anxiety, a GP observed it was rarely isolated:

*I mean it’s unusual to have* [health anxiety] *as a pure problem and then there not be an element of anxiety around anything else in the rest of their lives.* (GP4)

#### Stigma

Patients would often use physical symptoms to test the waters before they felt comfortable enough to ‘reveal’ their feelings of anxiety which could be a barrier to open discussion of symptoms:

*I think there again I start from a position of assumption that people generally will want to hide the psychological and offer you up the physical first for- and I put that down to stigma in society and their own stigma of themselves* (GP3)

#### Time constraints

The consultation time (10 min for most primary care practices in England), could be a barrier to understanding the true nature of the problem, particularly with new and anxious patients:

*the major challenge with that is that you’ve got a ten minute time slot to try, in theory, to get information, examine, document, it’s an awful lot to get done in ten minutes. It’s an unrealistic timeframe really and the clear risk, if you don’t know the patient and they’re not familiar with you and …they’re anxious* (GP4)

Time restrictions can also be a barrier to understanding complicated lives and situations:

*there’s mental health issues, unpleasant life events, single mum stress, abusive partnerships, there’s a kind of off the peg anxiety that you’re going to attach to that …So (laughs) ‘tell me how you’re going to cope with the rest of your life ‘cos I’m about to draw a line under our involvement’ so I find that quite difficult, jumping in on people who have got very complex lives.* (GP3)

All patients were aware of the time limits placed on the consultation and often expressed sympathy for the strain it placed on GPs. However, it also made it more difficult for patients who needed a bit of time to talk about their problems and symptoms associated with anxiety:

*GPs haven’t got enough time… so it’s being unreasonable on my part if I were to say ‘look I want to spend more time and talk because I can isolate this and I can isolate that, and my physical symptoms and my emotional problems’ but I know there is a shortage of money and there’s a shortage of time* (Female 65–75 ID)

#### Gender/age

Compared with women, older men did not talk about the impact of their anxiety symptoms or talk much – if at all - about the symptoms themselves within the consultation. For older, male participants, strongly held beliefs about self-reliance could inhibit any open discussion:

I *Would you ever chat to the GP about feeling anxious?*

*P I don’t think so. Just it’s me. That’s something I have to do myself*. (Male 65–75 ND)

*I’ve never been a forthcoming person… most of my life looked after myself and that’s what I am. What they call it? A loner. (laughs)* (Male 65–75 ID)

A commonly held belief among the women was that it was harder for men to talk about their feelings. Indeed, all bar one of those who openly discussed anxiety were women and aged between 22 and 44 years.

### Techniques to overcome barriers

#### Patients: choosing a GP

The patients who had openly discussed anxiety in their consultation all had previously diagnosed anxiety or anxiety states. All the participants in this group had seen the GP before and hadchosen to see that GP again because of their perceived positive qualities:

[The Dr] *was amazing on the phone and so kind and so understanding…I’ve seen her maybe twice over that time since then and she remembered… I picked her because I felt confident that she would understand where I was coming from and that I stood a good chance of her being reasonable and listening to me* (Female 35–50 OD)

The language of choice here is salient; to choose the right GP to consult about symptoms can make a difference between being truly heard, or not.

#### GP: repeat appointments

GPs often bring patients back for a further appointment to discuss anxiety or mood separately from their presenting problem. This ‘bridging’ from one consultation to another could be used as a way of keeping the conversation/rapport going with the patient to ensure best care and breathing space for the GP to reflect on possible care, or to gain more information if the patient is new:

*If it’s a new person and you’ve not met them before then even more so I think, to pick up on little things and say ‘ It doesn’t mean that we have to talk about it today but perhaps we can chat a bit more about your mood or depression or we can talk a bit more about the medication next time'* (GP2)

*Sometimes you also just need to go away and think about it. I don’t always have a solution which is why I say well actually we’ll pick this up in two weeks’ time.* (GP1)

Nonetheless, there is ambivalence about bringing patients back again related to the GP role and skills as well as what is possible given the time limits and the needs of other patients:

*It’s difficult. As a GP you don’t want to become a therapist... then you start to open things up too much then - you don’t have enough time to contain it in the consultation and sometimes you don’t have the skill to be able to really, you just leave someone more emotionally fragile than if you leave something* (GP1)

#### GP: identifying the priority problem

All the GPs described a range of similar techniques they used to facilitate the patient and themselves within the consultation (Table 3):

**Table 3:** GP technique to facilitate discussion of anxiety

	Examples used by GPs in interview
Open questioning	*start with a very open question to your consultation… not kind of hone down on anything too quickly because then it allows them to direct us to what they feel may be important*
Physical observation	*Rapid speech, agitation, wringing of the hands, whether they’re tearful, how they’re talking*
Attending to lists	*people will come to the end of their three or four things and mental health is one of them*
Asking about anxiety in key conditions	*if there’s a physical issue for example bowel problems or sleep problems or palpitations then you do tend to ask about the stress and anxiety and mood*

## Discussion

### Summary

Discussions about anxiety are influenced by a range of barriers and facilitators for patients and GPs. These often mirror each other.

In this study the main facilitator for the open discussion of anxiety is the rapport between doctor and patient. A good relationship was felt to be promoted by continuity of care and consists of attentive, empathetic and non-judgemental listening.

The main barriers to discussion are the attribution or acknowledgment of symptoms, co-morbidities and time constraints. How patients attribute their anxiety symptoms – especially physical symptoms – influences whether they are acknowledged as anxiety. Physical and psychological co-morbidities can mask symptoms, with depression and health anxiety often being conflated with anxiety. Time constraints and the role of the GP can conflict with the needs of the patient with anxiety. The tension between exploring a complex problem, managing elicited feelings and agreeing on a management plan within 10 or even 15 min was difficult and at times felt unworkable for both patients and GP.

GPs use a number of techniques within the consultation to facilitate discussion of anxiety. They also use the structural technique of repeat appointments to make more time and create some continuity of care by allowing the patient/GP relationship to develop. Participants often overcame barriers by choosing a particular GP who they felt would listen to their concerns about their symptoms.

However, anxiety may not be the most important problem for the patient *at that time*. Patients may have other, more urgent priorities or feel able to cope with quite high levels of anxiety. Socio-demographic, interpersonal and structural-level factors are also relevant to the aetiology of anxiety and can influence help-seeking behaviour.

There were indications that age and gender can play a role in whether or not anxiety is discussed. This study tended to support the cultural norm that women are more willing to talk about their psychological concerns. People below the age of 50 were also more likely to talk about their symptoms. In comparison to more recent ideas about the benefits of openly discussing mental health, it is likely that stoicism and silence is more ingrained in a certain cohort of older men.

Prioritisation of the physical over the psychological in the primary care consultation is a complex phenomenon and can arise in several ways. There was some evidence of a structured sequential approach to diagnosis on the part of the physician, with a need to exclude the physical first. There may be a reluctance on the part of the patient to mention the psychological dimension because the patient fears that their symptoms may be dismissed as ‘all in the mind’.

Threats to the GP/patient rapport and - especially from the GP perspective - continuity of care, can create a barrier to the conversation around anxiety. Mergers of several GP practices to provide more choice and swifter service to patients can hinder the very continuity of care GPs require to provide the best care for their patients; particularly patients with unacknowledged anxiety.

### Strengths and limitations

This study was confined to patients who had routine appointments in two Bristol practices. Four interviews with participants could not be arranged and thus their voices are missing from the data.

Participating GPs were self-selecting and more likely to describe themselves as interested in mental health. Three out of four GPs were in their 30’s which does not reflect the age distribution of GPs. A further study examining views of a wider group of GPs would be a welcome addition to understanding how anxiety is discussed and managed in primary care.

GPs and patients were informed that the study was about understanding how physical and psychological symptoms were prioritised in primary care. Anxiety was not specifically mentioned so as not to bias the primary care encounter. Patients and GPs were fully informed as to the study aims at the end of their interviews. Consequently, we would not expect the consultations to be biased in terms of discussing anxiety.

Qualitative methods are not intended to be generalizable but to represent the participant accounts under investigation as faithfully as possible.

The use of data from multiple sources: self-report questionnaires, video, patient and GP interview data all give a fuller understanding of the phenomena in question.

### Comparison with existing literature

Previous studies or reviews on the under-recognition of anxiety and depression in primary care [5, 12, 16, 17] have tended to focus on the clinicians’ experience and, often, with the emphasis on depression. Findings from our study – with a focus on anxiety - support several of the earlier results including: patients often have somatic presentations; those that attribute a psychological cause are more likely to be recognized; and those who normalize their symptoms are less likely to be identified [16].

As stated above, there was some evidence in our study that GPs and patients would initially prioritise physical symptoms. However, this did not mean that physical symptoms were always prioritised overall; they were often considered something to be negotiated before discussion of psychological symptoms could take place. Wallace [19] proposed prioritising the treatment of depression in consultations, the evidence suggesting that this enables patients to better manage their chronic physical conditions. It is possible that where anxiety is present with co-morbidities, prioritising could be similarly employed.

Previous studies investigating barriers or facilitators to disclosing mental health problems from the patients’ point of view, found that good communication and continuity of care were perceived to be integral to disclosure of mood problems [10, 15, 18] (again, mostly depression) whilst barriers included the perception of not being listened to, the prioritising of physical symptoms by the GP, stigmatisation and lack of time.

Similar barriers and facilitators were found in the disclosure of anxiety symptoms but these views were present with more or less emphasis from both the participants *and* the GP perspective. Time constraints were considered a major barrier for the clinicians in addressing anxiety while more significant barriers described by participants were the attribution and aetiology of their symptoms. There was some evidence that stigma associated with mental health is lessening, in line with recent public health messages, particularly for the younger cohort in the current study. However, there are indicators that these messages are not necessarily absorbed by some of the older, male participants.

It has been reported that primary care practitioners can feel unjustly criticised, with cross-sectional research into the prevalence of unrecognised depression and anxiety not accurately reflecting the complexities and longitudinal nature of recognition in primary care [16]. Despite the cross-sectional design of the current study, eliciting data from several sources addressed these concerns by highlighting the on-going nature of the process in recognising and discussing anxiety in primary care. The current findings show the importance of addressing the co-construction of anxiety by both sides of the doctor/patient dyad. Primary care consultations are a discussion, and the complicated aspects of anxiety are negotiated by both parties over time.

## Conclusion

These findings are an addition to the evidence base of patients experiences of anxiety and the discussion of symptoms in primary care; evidence that is lacking compared with the depression literature.

This cross-sectional study indicates that the discussion around anxiety is a process; it is not a simple matter of patients not disclosing or GPs not detecting anxiety symptoms. It can be hypothesised that this on-going process is negotiated between the GP and the patient and is influenced by a range of barriers and facilitators. GPs overcome barriers to the open discussion of anxiety by bridging patient consultations; bringing patients back for further appointments fosters continuity of care, and using prioritizing techniques enables them to identify anxiety symptoms which may not be acknowledged by patients. Future research could investigate this process using a longitudinal study design and by recruiting a more diverse range of GP surgeries and GPs.

Our findings indicated that GPs should be mindful that when consultations with patients involve psychological problems - in particular depression and health anxieties – that they can mask generalized anxiety symptoms. This is particularly important as patients with both depression and anxiety tend to have a worse prognosis. We acknowledge that health anxiety may be a feature of generalised anxiety. However, it can remain a narrow focus in the clinical situation and lead to a pattern of investigation of physical symptoms and reassurance. This it itself can be a barrier to a broader exploration of anxiety symptoms and their impact on the patient’s life.

Finally, there is further work to be done on public health messages of open discussion about mental health problems, particularly to reach older men.

## Additional files

Additional file 1: Topic guide. (DOCX 41 kb)

Additional file 2: Table of extra sociodemographic characteristics of study participants. (DOCX 15 kb)

Additional file 3: Symptoms attributed to anxiety by patients or GPs. (DOCX 17 kb)

Additional file 4: Co-morbidities presented in consultation. (DOCX 22 kb)

## Data Availability

The data that support the findings of this study are not available for sharing, as participants did not consent to data being shared beyond the research team and/or project.

## Abbreviations

CRN: Clinical Research Network
GAD-7: a 7-point self-reported questionnaire for screening and severity measuring of Generalized Anxiety Disorder.
GP: General Practitioner [in primary care]
HAI: Health Anxiety Inventory
IAPT: Improving Access to Psychological Therapies
ID: [anxiety] implicitly discussed
MB: Maria Barnes, Main Researcher
ND: [anxiety] not discussed
OD: [anxiety] openly discussed
PHQ-9: a 9-question from the Patient Health Questionnaire given to patients in a primary care setting to screen for the presence and severity of depression
UK: United Kingdom
UPPS Study: Understanding how physical and psychological problems are prioritized in primary care Study
