# Supplementary material for: Prioritising physical and psychological symptoms: what are the barriers and facilitators to the discussion of anxiety in the primary care consultation?
Source: BMC Fam Pract. 2019 Jul 27;20:106. doi: 10.1186/s12875-019-0996-6 (PMC6660691; doi:10.1186/s12875-019-0996-6)
Supplement: Supplementary file 1 — Topic guide. (DOCX 41 kb) [file 12875_2019_996_MOESM1_ESM.docx]

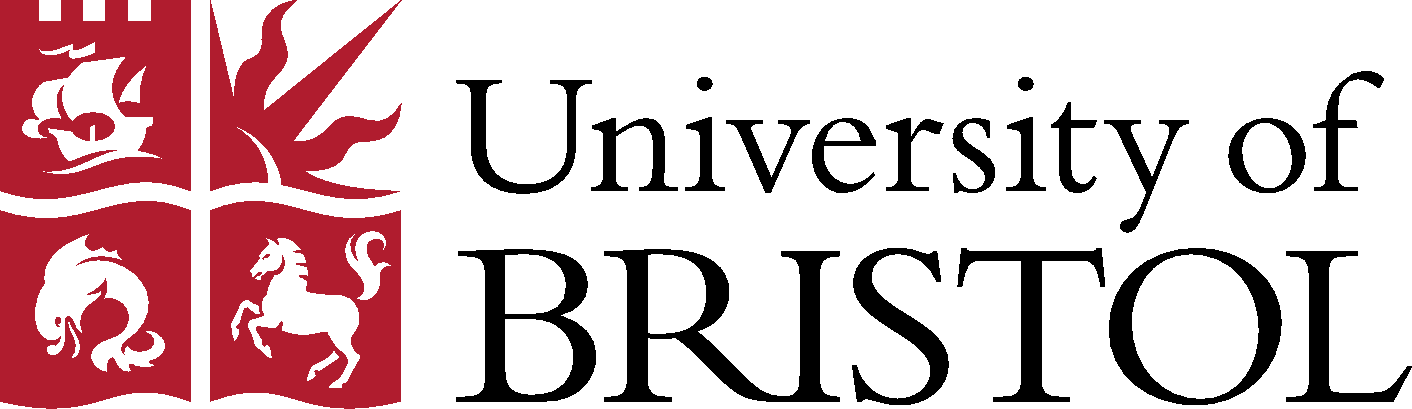
 IRAS Ref: 237902

**Understanding how physical and psychological symptoms are prioritised**

**in the GP consultation**

**TOPIC GUIDE FOR PARTICIPANTS**

Please tell me anything you can recall about your consultation ** days ago

- What did you talk about?
- How did you describe your symptoms?
- How long have you had symptoms?
- Was there more than one thing you discussed?
- Did you feel the physical or psychological symptoms were the priority?
- How did the GP respond?
- What sort of treatments did you talk about?
- What were your feelings about that?
- Was there anything you didn’t talk about (but wanted to)? (i.e. anxiety and if not why not)
- What made it difficult or easy to talk about?
- What would help?

*Explain focus of study as anxiety*

- Ask about GAD-7 as a prompt to discussion with GP

Please tell me what you did about your symptoms after seeing your GP til now?

What have you done in the past about your symptoms?

Have you considered seeing a GP previously about your symptoms and not done so? Why?

Were your symptoms noticed by family or friends?
