# Supplementary material for: Prioritising physical and psychological symptoms: what are the barriers and facilitators to the discussion of anxiety in the primary care consultation?
Source: BMC Fam Pract. 2019 Jul 27;20:106. doi: 10.1186/s12875-019-0996-6 (PMC6660691; doi:10.1186/s12875-019-0996-6)
Supplement: Supplementary file 2 — Table of extra sociodemographic characteristics of study participants. (DOCX 15 kb) [file 12875_2019_996_MOESM2_ESM.docx]

Appendix 2 – Table of Extra Sociodemographic Characteristics of Study participants

|  | **All patients** | **Those interviewed** |
| --- | --- | --- |
|  | **(n = 160)** | **(n = 17)** |
|  |  |  |
| Age: mean (SD)† | 53.4 (19.6) | 47.4 (16.8) |
| Female: n (%)~ | 82 (52.2) | 10 (58.8) |

| ***Home ownership status: n (%)*⁺** |  |  |
| --- | --- | --- |
| Own home | 99 (63.5) | 7 (41.2) |
| Rent (private or local authority) | 44 (28.2) | 8 (47.1) |
| Other | 13 (8.3) | 2 (11.8) |
| ***Highest educational qualification: n (%)*ˆ** |  |  |
| O-levels/CSEs/GCSES/apprenticeship | 26 (16.5) | 2 (11.8) |
| AS/A-levels/advanced diploma/degree/higher degree/diploma | 74 (46.8) | 9 (52.9) |
| Professional qualifications/Other vocational/work related | 42 (26.6) | 3 (17.7) |
| Overseas qualifications | 4 (2.5) | 2 (11.8) |
| No Qualifications | 12 (7.6) | 1 (5.9) |
| ***Marital status: n (%)ˆ*** |  |  |
| Single | 35 (22.2) | 5 (29.4) |
| Married/civil partnership/cohabiting | 99 (62.7) | 8 (47.1) |
| Divorced/separated/widowed | 24 (15.2) | 4 (23.5) |
| ***Employment status: n (%)ˆ*** |  |  |
| Employed/self-employed | 84 (53.2) | 8 (47.1) |
| Retired | 58 (36.7) | 5 (29.4) |
| In full-time education | 6 (3.8) | 2 (11.8) |
| Looking after home or family/unemployed | 5 (3.2) | 2 (11.8) |
| Unable to work due to illness/condition | 5 (3.2) | 0 (0) |
| White/white british/white other: n (%)ˇ | 143 (93.5) | 13 (92.9) |
| ⁺*n = 156* |  |  |
| *ˆ n = 158* |  |  |
