# Supplementary material for: Prioritising physical and psychological symptoms: what are the barriers and facilitators to the discussion of anxiety in the primary care consultation?
Source: BMC Fam Pract. 2019 Jul 27;20:106. doi: 10.1186/s12875-019-0996-6 (PMC6660691; doi:10.1186/s12875-019-0996-6)
Supplement: Supplementary file 3 — Symptoms attributed to anxiety by patients or GPs. (DOCX 17 kb) [file 12875_2019_996_MOESM3_ESM.docx]

| **Appendix 3: Symptoms attributed to anxiety by patients or GPs.** | |
| --- | --- |
| Shortness of breath  Hives  Insomnia  Feeling sick  Shaking  Being unable to relax  Worrying constantly  Sweating | Hyperventilation  Racing heart  Crushing of chest  Sweating  Headaches/migraines  Stress  Irritable bowel syndrome |
