# Supplementary material for: Prioritising physical and psychological symptoms: what are the barriers and facilitators to the discussion of anxiety in the primary care consultation?
Source: BMC Fam Pract. 2019 Jul 27;20:106. doi: 10.1186/s12875-019-0996-6 (PMC6660691; doi:10.1186/s12875-019-0996-6)
Supplement: Supplementary file 4 — Co-morbidities presented in consultation. (DOCX 22 kb) [file 12875_2019_996_MOESM4_ESM.docx]

| **Appendix 4: Co-morbidities presented in Consultation** | |
| --- | --- |
| Participant Group | Co-Morbidities presented in Consultation |
| Openly Discussed [OD] | Diabetes  Bi-polar/panic attacks/agoraphobia/claustrophobia  Hair loss (heavy periods: anaemia, fibroids, menopause)/Sleeplessness  Irritable Bowel Syndrome  Multimorbidity |
| Implicitly Discussed [ID] | Depression/Gout/Recent pneumonia  Spot near eye  Idiopathic lung fibrosis  Depression  Sleeplessness/Depression/alcohol abuse  Shortness of breath/dizziness/shoulder pain |
| Not Discussed (ND] | Back pain  Diabetes/Heart condition (CHECK)  Tired all the time ? Chronic Fatigue Syndrome  Post-operative review for diverticulitis  Hip and pelvis pain  Painful foot  Adult acne |
